# An Improved Pattern Synthesis Iterative Method in Planar Arrays for Obtaining Efficient Footprints with Arbitrary Boundaries

**DOI:** 10.3390/s21072358

**Published:** 2021-03-28

**Authors:** Aarón Ángel Salas-Sánchez, Cibrán López-Álvarez, Juan Antonio Rodríguez-González, María Elena López-Martín, Francisco José Ares-Pena

**Affiliations:** 1 ELEDIA@UniTN, Department of Information Engineering and Computer Science (DISI), University of Trento, 38122 Trento, Italy; aaronangel.salas@usc.es; 2 CRETUS Centre, Department of Applied Physics, University of Santiago de Compostela, 15782 Santiago de Compostela, Spain; cibran.lopez.alvarez@rai.usc.es (C.L.-Á.); ja.rodriguez@usc.es (J.A.R.-G.); 3 CRETUS Centre, Department of Morphological Sciences, University of Santiago de Compostela, 15782 Santiago de Compostela, Spain; melena.lopez.martin@usc.es

**Keywords:** antenna arrays, pattern synthesis, footprint patterns

## Abstract

In the present paper, an iterative technique devoted to reproducing efficient footprints with arbitrary boundaries for planar arrays is addressed. The methodology here depicted is based on exploiting the nature of the continuous aperture distribution by expressing it as a Fourier series of moderately high orders. In this manner, the resulting illumination boundary is defined by a target three-dimensional flat-topped pattern composed of stretching and shrinking modified circular Taylor patterns and the maximum order of the series to obtain a good reconstruction is determined by means of the iterative process. Examples and comparisons with the previous literature were conducted by analyzing square and rectangular contoured beams as test cases. Additionally, interesting potentials regarding space applications from a geostationary satellite are outlined by means of the EuTELSAT (European Telecommunications Satellite Organization) European coverage case study. In such a way, its numerical approach was analyzed by including subarray architectures and discussing improvements about dynamic range ratio of the excitations without critical power losses within the illumination region.

## 1. Introduction

In order to generate an efficient footprint pattern from a planar array antenna mounted on a geostationary satellite, the number of radiating elements of the antenna should be minimized, while the shape of the radiation pattern fits some desired bounds. Thus, the importance of a well-defined covering region relies on the ability to avoid non-desired interferences with other signals, as well as to improve the flexibility and minimize costs of the feeding network mounted on the space vehicle. A valid approach for reducing the number of radiating elements can be developed by means of the use of subarrays. Focusing on this last strategy, an increase in both mobility and simplicity of the radiating system can be achieved, while fewer radiating elements are needed at the expense of provoking the appearance of undesired moderately high lobes in the power radiation pattern (grating lobes).

Several years ago, an efficient procedure which represents a generalization to arbitrary footprints of the Elliott–Stern method [1] was devised by Ares et al. [2]. That technique exploits the synthesis of a stretched pure-real continuous aperture based on the modified circular Taylor methodology produced by setting as radius a value which is inversely proportional to the flat-top beamwidth boundary. In such a way, it discretizes the continuous aperture distribution by means of the strictly needed number of elements to reproduce the pattern (by dismissing the array elements outside of the boundary). This procedure has been implemented as input of an optimization based on the Fletcher–Power method [3], necessary to improve the results in terms of side lobe level (*SLL*). More concretely, reductions from −11.27 dB to −20 dB have been reported for an initial Taylor pattern with a nominal *SLL* of −25 dB. At the same time, it is worth highlighting the problems of the methodology with reproducing a footprint which perfectly fits the square contour of the main beam, because of the use of the zero order of the Bessel function of the first kind, something which is based on the φ-symmetry of the contributions to the pattern description. Attempts to improve the efficiency of antenna arrays obtained with this procedure have been reported in [4], where a strategy of deleting elements with low-amplitude excitations has also been developed. In that manner, this technique achieves significant reduction in the number of excited elements, the cost, and the dynamic range ratio $\left({\left|I\right|}_{max}/{\left|I\right|}_{min}\right)$ of the antenna array excitations for square and elliptical footprint patterns.

Reinforced improvements on the planar array performance for reproducing arbitrary footprint shapes have been analyzed by two works [5,6] exploiting the concept of spreading out real collapsed distributions [7]. More precisely, a process involving a simulated annealing algorithm [8] has been proposed in [5] for overcoming the problem of sticking in local minima (in contrast to the Fletcher–Power method), while a solution by means of singular value decomposition of an over-determined system of equations (involving more angular cuts for improving) has been proposed in [6].

Further descriptions on arbitrary footprint reconstructions from planar arrays have been analyzed in [9], where a method based on perturbing the bi-dimensional Woodward–Lawson technique [10] has been proposed.

The attempt made in [11] is also remarkable, proposing a control of shaped-beam patterns obtained from a uniform aperture amplitude and only adjusting the relative phases. At the same time, a technique which introduces complex excitations distributions into the problem of arbitrary footprint pattern reconstruction is reported in [12]. With a similar aim, a procedure exploiting the φ-symmetry by dividing the circular aperture in angular sectors and synthesizing different excitation distributions for each one using a set of Taylor roots is discussed in [13]. Phase-only control methods are also addressed in [14], where it allows the reconstruction of the radiation pattern with planar reflect arrays of a huge number of elements.

Another approach based on a two-stage technique for generating a precise footprint is proposed in [15]. In this work, a continuous aperture distribution that approximates the generation of a desired footprint pattern by a Fourier series is then sampled and their elements excitations are involved within a simulated annealing optimization for improving its performance. In the same line of the previous work, an efficient method for the synthesis of footprint patterns that combines Hankel transformation with Fourier analysis following angle-dependent homothesis (i.e., radial stretching and/or shrinking) of an axisymmetric Elliott–Stern pattern for a circular aperture is reported in [16].

In order to improve the precision of the reproduction of a desired footprint pattern obtained by a planar array, a two-stage array synthesis process in which an optimization of the array boundary is followed by an optimization of the elements excitations is depicted in [17]. For the improvement in scenarios with hundreds or thousands of elements, an efficient combination of the Woodward–Lawson and Orchard–Elliott–Stern roots optimization procedures [18] is suggested in [19].

Another alternative referred to in the literature [20] is based on a quasi-analytical synthesis of moderate and large arrays, which proposes the shaping of a desired footprint as a composition of several φ-symmetric circular Taylor patterns exhibiting flat-topped beams. In this same spirit, a method which synthesizes the desired footprint as a composition of a set of circular Taylor patterns appropriately weighted with the samples of the pattern obtained after angle-dependent homothesis of a continuous circular aperture distribution developed by the Elliott–Stern method is proposed in [21].

Additionally, previous studies in the body of the knowledge, which also face the generation of footprint patterns, can be highlighted. More precisely, works as [22] describe two approaches for generating flat-top footprint patterns: the first one deals with array patterns required to have equal phase in all directions of the shaped region, while the second one is suitable for patterns without phase requirements and formulated by means of a nonlinear problem. In other works, collapsed distributions can also be applied to arbitrarily chosen grids, as shown in [23], where a hexagonal-shaped antenna is implemented (without imposing the limitation of quadrantal symmetric beam patterns). A convex optimization based on a beam pattern synthesis method with antenna selection has been proposed in [24]. This method can achieve a main lobe and side lobes of arbitrary beamwidth and response ripple level. On the other hand, a technique based on an iterative algorithm devoted to optimizing the excitation of each radiating element by applying convex optimization is described in [25]. Despite the algorithm being simple and fast, it does not guarantee reaching the optimal result.

The aim of this paper is to develop a new technique, consisting of the reconstruction of a desired footprint pattern by addressing an iterative refinement of the continuous aperture distribution devoted to generating this theoretical target pattern by means of its expression as a Fourier series. The present method overcomes the performance of the method described in [2], since it exploits the general Fourier series expression of the aperture distribution and shares the same strategy to select the array elements for sampling the continuous aperture distribution.

As it is reported in the present work, this method enables the modification of the original φ-symmetric-shaped pattern onto a desired contour. In the present paper, advances of the conventional deterministic array pattern synthesis strategies are highlighted in terms of optimality of the array antenna shape according to the required specifications and contour of the desired footprint. In order to illustrate the performance of the technique for space application purposes, the contour of continental Europe is addressed. In such a way, the design here analyzed concerns the EuTELSAT (European Telecommunications Satellite Organization) W2A WideBeam European coverage requirements [19,20,21,26], where a well-fitted optimized footprint of a shaped-beam pattern is obtained by reproducing the Fourier coefficients of a target illumination footprint, composed by different scaled φ-cuts of an initial flat-topped pattern. Considering the efficiency of the antenna, the dynamic range ratio is reduced by eliminating non-crucial radiating elements [2,4] (those with low amplitude excitations) and by implementing strategies including subarrays [21]. In this last case, it is worth mentioning that, due to the spacing between the different subarrays, undesired grating lobes appear in the resulting radiation pattern, but they are inconsequential since they are generated at angular levels out of the illumination range of the Earth from a geostationary (GEO) satellite point of view.

## 2. Materials and Methods

Let us consider a practical application of a planar array, where a certain boundary condition in terms of illumination (for instance, the coverage of certain region on Earth) must be achieved. In this manner, the pattern is constrained to fit a particular shape. For this scope, implementation of a three-dimensional main beam with φ-dependence on the radiation far-field pattern shape becomes mandatory. In such a way, a solution in terms of a generalization of the technique developed by Taylor can be proposed, something in line with previous works in the literature [15,16,20,27].

Therefore, let us analyze an extension of circular Taylor distributions developed by Elliott and Stern [1]. According to this, the expression for producing a flat-topped beam from a pure real continuous distribution (although in [28] a multiplicity of solutions have been recently described, among all the solutions presented, just one of them represents the pure real approach of the aperture function and coincides with the one described in [1]) is given by

(1) $$F(u)=2\frac{J_{1}(\pi u)}{\pi u}\frac{\prod_{n=1}^{M}\left[1-\frac{u^{2}}{{\left(u_{n}+jv_{n}\right)}^{2}}\right]\times \left[1-\frac{u^{2}}{{\left(u_{n}-jv_{n}\right)}^{2}}\right]\prod_{n=M+1}^{\bar{n}-1}\left[1-\frac{u^{2}}{{\left(u_{n}\right)}^{2}}\right]}{\prod_{n=1}^{\bar{n}+M-1}\left(1-\frac{u^{2}}{\gamma_{1n}^{2}}\right)}.$$

where $\gamma_{1n}$ is the *n*-th root of the first order Bessel function of the first kind $J_{1}(\pi u),$ $\bar{n}$ the transition parameter (which defines the number of controlled side lobes of the pattern produced by the Taylor method), u=(2a/λ)sinθ and a the radius of the circular boundary of the aperture. The complex numbers $u_{n}\pm jv_{n}$ are the modified roots of the uniform function $J_{1}(\pi u)/\pi u$ necessary for providing a flat-topped beam pattern with controlled *SLL* and ripple level. In this framework, the φ-symmetry of the function was proposed as the initial assumption made by Taylor.

Previous works introduced a modified version of this Taylor technique by stretching this pure real-continuous aperture. More precisely, Ares et al. [2] proposed to synthesize a distribution with a boundary that is inversely proportional to the flat-top beamwidth on each φ-cut.

In the present case of study, the first stage of the methodology concerns generating a continuous aperture distribution K(ρ,β) addressing its theoretical relation with the far field radiation pattern. In the framework of this reconstruction, we can express the far field pattern as a Fourier series in φ with coefficients

(2) $$F(u,\varphi )=\sum_{n=-\infty }^{+\infty }e^{jn\varphi }F_{n}(u).$$

Thus, in accordance with the aim of the present paper, a function F(u,φ) can be composed by means of a combination of circular Taylor patterns (1) presenting different effective radii. More precisely, as is well-known, one can reproduce a footprint by adapting the radius of a certain pattern depending on the φ angle.

Therefore, the above-mentioned coefficients can be calculated, by means of a regular Fourier inversion of (2), as

(3) $$F_{n}(u)=\frac{1}{2\pi }\int_{-\pi }^{\pi }F(u,\varphi )\;e^{-jn\varphi }d\varphi .$$

These coefficients can be expressed by means of the aperture distribution for each angle by the transformation [7]

(4) $$F_{n}(u)=\int_{0}^{\pi }pg_{n}(p)J_{n}(up)dp.$$

where

(5) $$g_{0}(p)=\frac{2}{\pi^{2}}\sum_{m=0}^{\infty }\frac{F(\gamma_{1m})J_{0}(\gamma_{1m}p)}{J_{0}^{2}(\gamma_{1m}\pi )}$$

and

(6) $$g_{n}(p)=-\left(\frac{2}{\pi^{2}}\right)\sum_{m=1}^{\infty }\frac{F_{n}(\gamma_{nm})J_{n}(\gamma_{nm}p)}{J_{n-1}(\gamma_{nm}\pi )J_{n+1}(\gamma_{nm}\pi )}n\neq 0.$$

where p=πρ/a and generally $\gamma_{mn}$ correspond with the *m*-th zero of the *n*-th order Bessel function of the first kind $J_{n}\left(\pi \gamma_{mn}\right)=0$.

Therefore, once $g_{n}(p)$ is obtained, the planar aperture distribution can be determined through the Fourier series [7] as

(7) $$K(\rho ,\beta )=\sum_{n=-\infty }^{+\infty }K_{n}(\rho )e^{jn\beta },$$

where each coefficient term of the series can be expressed as

(8) $$K_{n}(\rho )=\frac{\pi }{2a^{2}}{\left(j\right)}^{-n}g_{n}(p).$$

**Figure 1:**
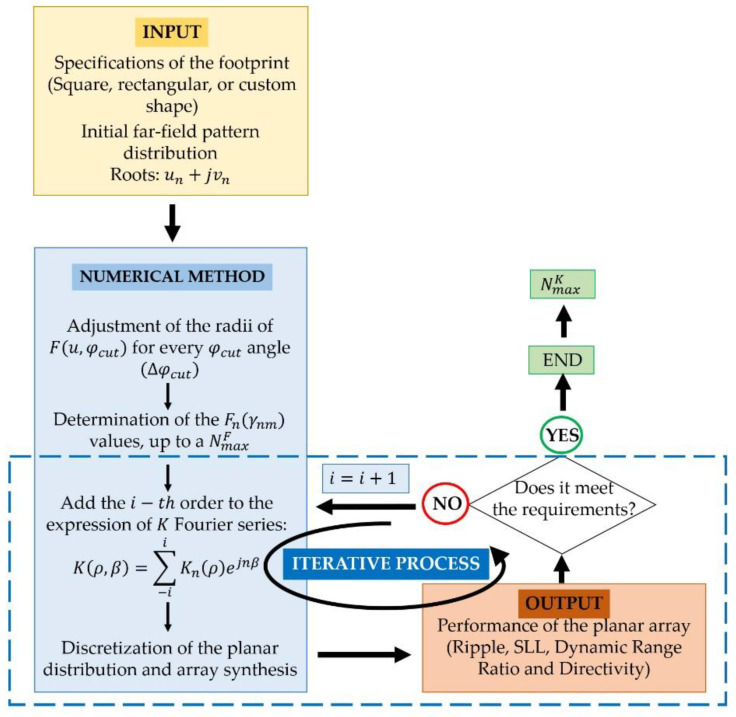
Conceptual flowchart of the procedure for synthesizing a certain footprint pattern.

Although these series generally do not truncate since their coefficients are obtained by means of (3), adopting a pattern composed from different scaled two-dimensional modified circular Taylor patterns (by means of their radii) for each one of its cuts, one can expect that they converge rapidly for practical apertures [7]. Based on this idea, an iterative process in which the performance of the method is evaluated by means of the reconstruction of the final far-field radiation pattern and a flowchart of the process is reported in Figure 1. Initially, the method determines the Fourier coefficients of the target function F(u,φ) by means of (3) up to a predetermined maximum number of orders $N_{\max}^{F}$ (in all cases, a good performance was achieved by setting this value to 50) by truncating the series. Then, through the iterative process depicted in Figure 1, the maximum $N_{\max}^{K}$ order for obtaining a good reconstruction through the aperture series (7) is determined by analyzing the results obtained after discretizing the aperture through the planar array with a rectangular lattice.

In this manner, the method described here represents an advance on the previous work devised in [1], since it represents a generalization to upper orders of the Fourier series in which the K(ρ,β) is determined (7). In order to illustrate the performance of the iterative process, the different results attending different maximum orders of the aperture distribution series to be discretized will be shown in the results section.

The second stage of the numerical method corresponds to a discretization of the obtained aperture distribution. Thinking about improving the development of this sub-process and based on the work of Hodges et al. [29], a rectangular layout of elements which fits the required aperture shape is sampled by performing an integrated strategy for obtaining the excitation coefficients. The sampling process here developed can be illustrated by means of Figure 6.14 in [7] or Figure 2 in [29]. Therefore, the excitation currents of the array factor can be obtained, and the expression of the array factor can be determined by means of

(9) $$F(\theta ,\varphi )=\sum_{l=1}^{N_{elem}}I_{l}e^{jk\left[x_{l}u(\theta ,\varphi )+y_{l}v(\theta ,\varphi )\right]}.$$

where $N_{elem}$ is the total number of elements of the array; $I_{l}$ are the relative excitation amplitudes obtained by the Hodges method [29]; k is the wavenumber; $\left(x_{l}{,y}_{l}\right)$ are the Cartesian coordinates of each array element; u(θ,φ)=sinθcosφ; and v(θ,φ)=sinθsinφ where θ and φ are the elevation and azimuthal angle, respectively.

In the particular case of the square contour, and for developing a fair comparison with the performance of the previous work of Ares et al. [2], octant symmetry in (9) was imposed. Therefore, the expression of the array factor of the square lattice can be modeled by expressing the array factor through the simplified expression for quadrantal symmetry as $F(\theta ,\varphi )=4\sum_{m=1}^{M_{d}}\sum_{n=1}^{N_{d}}I_{mn}\cos\left[kx_{m}u(\theta ,\varphi )+ky_{n}v(\theta ,\varphi )\right]$ where $M_{d}$ and $N_{d}$ are the limits of the number of elements in the *x*-axis and *y*-axis, respectively. In such a way, the octant symmetry can be guaranteed by imposing $I_{nm}=I_{mn}$ for the elements inside the mandatory boundary for generating a square contoured beam pattern. Otherwise, the elements out of the aperture boundary limits are switched off.

As shown in [2], the planar aperture necessary for generating a footprint pattern whose contour fits a square boundary results in a quatrefoil-shaped aperture distribution, since it is the shape which fits the flat-top beamwidth. Thus, as the first approach to the square contoured performance, a comparison with the work of Ares et al. [2] was conducted. Then, a generalization to arbitrary shapes of the three-dimensional pattern is conducted and even cases which present a clear asymmetry on the antenna array factor expression are addressed. Therefore, a rectangular boundary is included by generalizing the method to a custom shape as input. Finally, a numerical application concerning the illumination of continental Europe from a geostationary satellite was analyzed.

**Figure 2:**
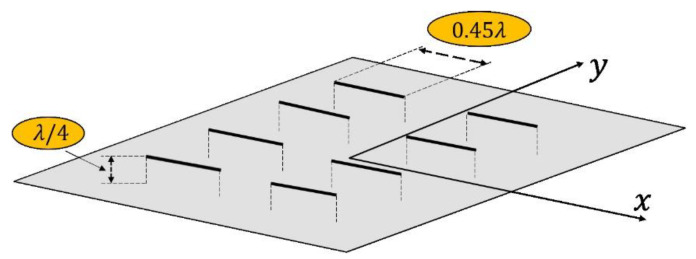
Detail of an array of center-fed electric dipoles set λ/4 above an infinite ground plane introduced in the study by means of its element factor [7,30].

Additionally, in all cases, the element factor of a dipole above a ground plane [7,30] is introduced in order to analyze the impact of a real element within the planar array. In such a way, Figure 2 shows a sketch of the proposed implementation. The dipoles are center-fed and have a length of 0.495 λ (for preventing contacts overlapping at 0.5 λ spacing). These dipoles are placed at a distance of λ/4 in front of a large conducting plane, and radiation occurs into the half space z>0.

## 3. Results

In line with the developments reported in the previous section, different results regarding the performance of the methodology will be analyzed in the following subsections. As first step, a comparison with a previous technique of the literature [2] is addressed by evaluating the performance of the present study and comparing it with the performance of a stretched aperture distribution $g_{0}$ for modified Taylor patterns depending on the φ-cut for producing a general g(ρ,β) with a boundary $\rho_{\max}(\beta )$.

Then, a generalization to arbitrary boundary shapes is developed by adjusting the radius to each pattern cut and without imposing any type of symmetry of the pattern. 

**Figure 3:**
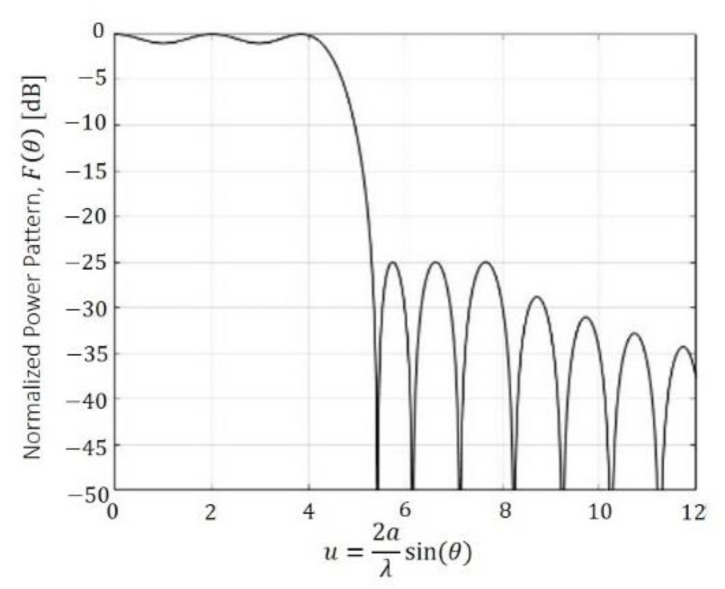
Shaped pattern with a ripple level of ±0.5 dB  and SLL=−25 dB, generated by a pure-real distribution.

As an initial pattern to compose all the footprints, a pure-real flat-topped beam pattern by means of (1) and with SLL=−25 dB, $\bar{n}=6$ and M=2 (as in the Elliott–Stern example [1]) was selected. The pattern is illustrated in Figure 3.

This type of shaped pattern was set according to comparison issues following previous works in the literature. It is worth highlighting that other examples of shaped-beam patterns could be also addressed in this development: examples with more or less ripple cycles in the main beam region. However, regarding this idea, in case of a higher number of cycles, an extreme variability of the continuous aperture distribution was proven and, therefore, more problems regarding dynamic range ratio $\left(|I{|}_{\max}/|I{|}_{\min}\right)$ on the resulting discretized antenna can be pointed out. Otherwise, cases presenting just one ripple cycle were tried and they will force the appearance of smoother slopes in the transition phase of the shaped pattern by provoking an enlargement of the width of the main beam region. Additionally, continuous aperture distributions based on complex variables, as in the example discussed by Elliott and Stern in [31], or the entire multiplicity of solutions reported in [28], can be involved in the present methodology, but the initial footprint related to these studies would be complex. In such a way, the coefficients of the Fourier series obtained by means of the method will present a phase value compatible with a circular boundary. Therefore, higher ripple levels due to these limitations of the methodology in managing the pattern phases can be expected.

### 3.1. Square Footprint: Quatrefoil Shape of the Antenna

Let us consider the same test case analyzed in [2], with a square footprint of 40°×40° approximately (i.e., with a continuous aperture radius of a=6λ). Therefore, as the first step of the methodology, the same quatrefoil shape proposed in this work was implemented. In the following sections, the different results which characterize the solution are analyzed.

#### 3.1.1. Reconstruction of the Far-Field Pattern by Means of the Fourier Series

**Figure 4:**
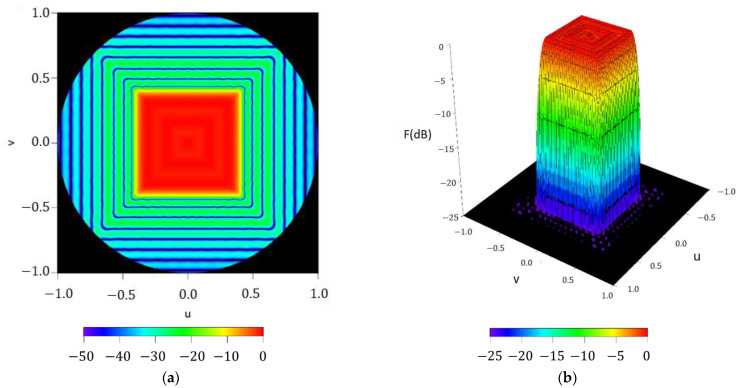
Reconstructed pattern of the test case of a square boundary of approximately 40°×40° by means of the obtained Fourier coefficients in (2): (**a**) interpolated image with a threshold level set at −50 dB; (**b**) surface plot with a threshold level set at −25 dB;

In order to check the performance of the methodology and to set the maximum order required to guarantee good pattern reconstruction, the radiation pattern F(u,φ), reconstructed by means of the Fourier series with $N_{\max}^{F}=50,$ is reported in Figure 4. A pattern with a ripple level of ±0.52 dB and SLL=−24.52 dB is obtained. At the same time, it is worth highlighting how the pattern function reproduces the square contoured shape, not only in the region of emission, but also in the side lobe region where the pattern nulls are arranged in a square shape as well.

#### 3.1.2. Convergency Study of the Results. Performance of the Method

**Figure 5:**
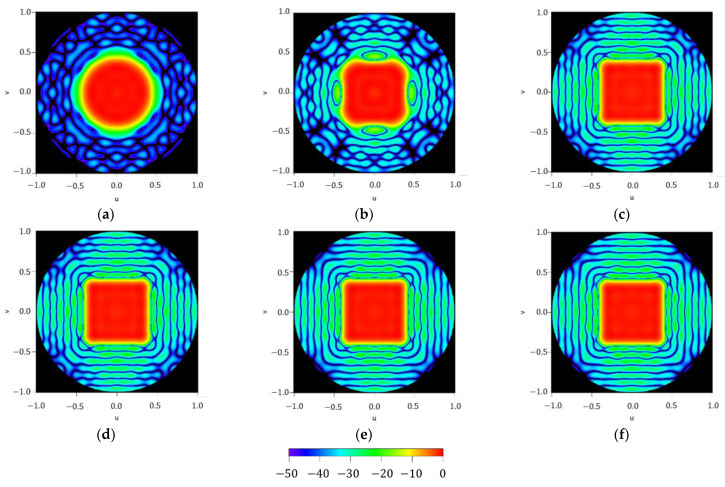
Figure series illustrating the iterative process devoted to generating a squared footprint by increasing the maximum order of the aperture distribution series $N_{\max}^{K}$ described in (7): (**a**) $N_{\max}^{K}=0,$ (**b**) $N_{\max}^{K}=4,$ (**c**) $N_{\max}^{K}=8,$ (**d**) $N_{\max}^{K}=12,$ (**e**) $N_{\max}^{K}=16,$ and (**f**) $N_{\max}^{K}=20$. All the interpolated plots described here have a threshold level set at −50 dB for the normalized far-field power.

To illustrate the performance of the iterative method described here, results regarding the different iterations on the maximum order reached by the approximated Fourier series of the aperture distribution are reported in Figure 5. It can be noted how the methodology starts from an almost circular shape of the footprint (at n=0) and then, by adding more orders to the series (more precisely, in case of a square contour, only the order proportional to four has no null contribution to the iterative process), the technique improves the shape of the resulting pattern to finally reproduce the required footprint (as can be seen in the flowchart illustrated in Figure 1). Regarding the three final steps of the iterative process, SLLs of −21.47 dB, −22.31 dB and −22.96 dB can be referred to, as well as the improvement in terms of ripple level, which falls from ±0.78 dB to ±0.75 dB and finally to ±0.71 dB.

#### 3.1.3. General Results of the Iterative Method

**Figure 6:**
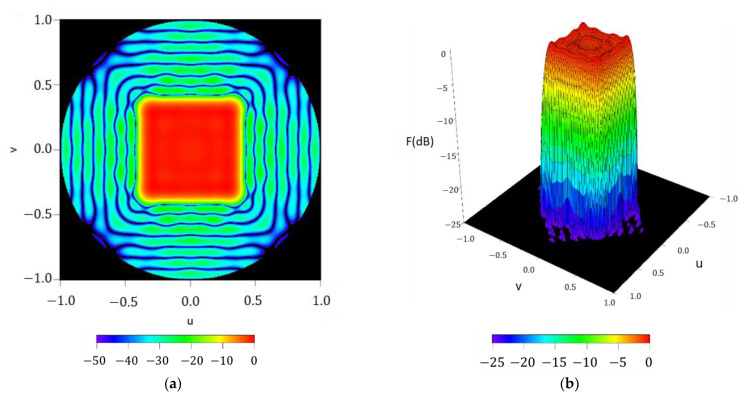
Reconstructed pattern of the required footprint for the case of a squared boundary of 40°×40° by means of a discretized array antenna: (**a**) interpolated image with a threshold level set at −50 dB; (**b**) surface plot with a threshold level set at −25 dB.

**Figure 7:**
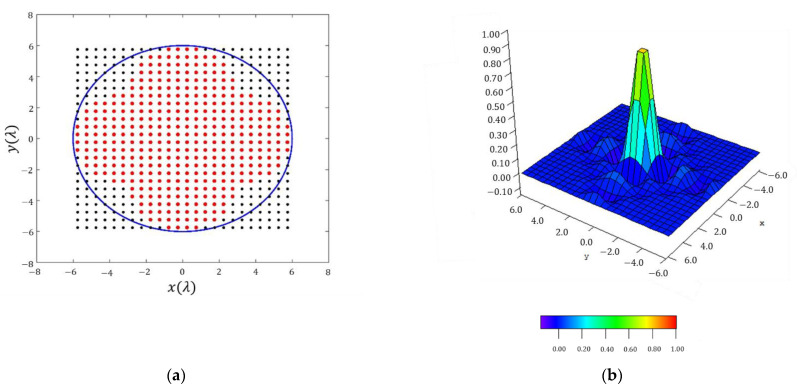
Discretized array antenna which generates the pattern of Figure 6: (**a**) antenna array configuration (the red dots represent the elements switched ON) within a rectangular lattice and for a maximum radius of 6 λ. (**b**) Normalized excitation currents of the array antenna.

In this section, a description of the results of the iterative method is provided. In particular, the resulting pattern (reported in Figure 6) was reached by expressing the aperture by means of a Fourier series with maximum order $N_{\max}^{K}=20$ and refers to a ripple level of ±0.71 dB, SLL=−21.96 dB and a directivity of D=13.96 dBi. In this final result, it can be confirmed how the array antenna pattern nulls are arranged in a square contour (confirming the behavior of the reconstructed pattern reported in Section 3.1.1) not only in the main beam region but also in the region of the sidelobes. This fact gives an idea about how this technique outperforms the methodology envisaged in [2], which presents a shape for the region of side lobes with circular symmetry. The planar array antenna presents 49 elements in the first octant, i.e., a total number of 368, which present a dynamic range ratio $\left(DRR={\left|I\right|}_{\max}/{\left|I\right|}_{\min}\right)$ of 2633 (see Figure 7).

In order to understand the improvements of the present methodology by means of the fitted shape of the resulting array, it is worth highlighting that, for a conventional square grid and a square boundary, 576 elements would be necessary to produce the pattern. On the other hand, considering a circular boundary, 448 elements would be necessary. Therefore, for the particular case of the quatrefoil shape of the antenna, a relative decrease of −36.10% and −17.86% could be reported, respectively.

In addition to the advances in performance reported by the present methodology, an alternative path of improvement to the work developed by Ares et al. [2] can be performed by increasing the order of the aperture distribution discretized by means of (6) in [2]. More precisely, the technique implemented by Ares et al. reports a pattern with SLL=−11.27 dB and a ripple level of ±0.715 dB. Then, by increasing the orders of the aperture distribution and adding four iterations, a resulting pattern with SLL=−21.95 dB and a ripple level of ±0.71 dB is generated. Therefore, an improvement of the results by means of the introduction of the present iterative strategy in the description of the aperture distribution in [2] can be highlighted.

Furthermore, to understand the impact of the inclusion of a real radiating element in the array structure, the case sketched in Figure 2 was introduced. In such manner, the directivity level of the case was raised up to 14.02 dBi and the SLL and ripple level were slightly improved to −22.03 dB and ±0.70 dB. Therefore, little improvements of the performance of the array pattern can be outlined.

**Figure 8:**
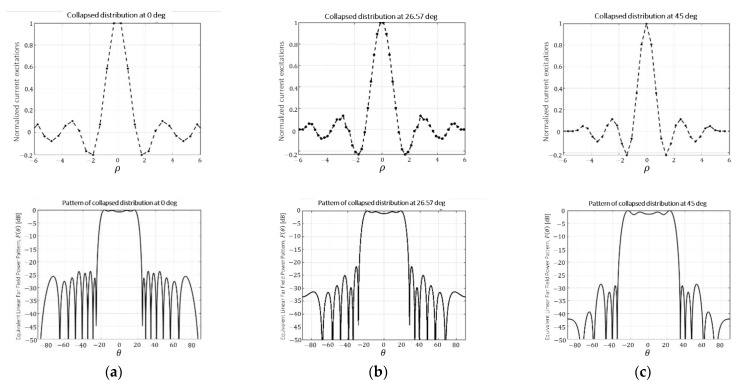
Aperture distributions and far field patterns of different collapsed cuts of the planar array whose three-dimensional pattern is reported in Figure 6: (**a**) 0°; (**b**) 26.57°; (**c**) 45°. These figures illustrate the effective size of the antenna on each one of the cuts.

In order to understand the effective dimensions of this antenna regarding the different antenna pattern cuts, the collapsed distributions at three different angular cuts (0°, 26.57°, and 45°, in the same spirit of [5,6]) are reported in Figure 8. In such a way, the reader can be aware of the effective size of a planar array, which (at some angles) is greater than the real size of the antenna. This development will give an idea about the essential number of elements necessary to reconstruct a certain footprint from a planar array antenna. For instance, the case at 45° represents the greatest expression of the above-mentioned misalignment between the real and the effective size of the antenna. More precisely, the geometrical limitations of the planar array established by the quatrefoil boundary at this φ-cut is approximately 4.24λ, while, analyzing the collapsed distribution at this same cut, an equivalent linear array with a semilength of 5.25λ is reported.

On the other hand, thinking about a practical application of this shape, it could be used to reconstruct a pattern devoted to fit a square footprint of a very restrictive size such as 1°×1° for instance (something which could be interesting for certain limited regions present on Earth, as the central region of the Iberian Peninsula, because it roughly approximates such a region). In such case, it is worth highlighting that the required antenna array for this scope becomes unfeasible in practice. The idea for understanding this proposal as non-realizable is based on the reason that it would need 71,108 radiating elements per octant (i.e., around 568,000 elements for the entire array antenna).

### 3.2. Generalization to Different Footprint Shapes: Rectangular Boundary

In order to address different footprint shapes, a process devoted to modifying the radius of each aperture distribution for generating the pattern on each angular cut for obtaining the required beamwidth was developed. In such a way, a rectangular footprint with dimensions of 20°×40° was addressed to illustrate the performance of the method.

#### 3.2.1. Reconstruction of the Far-Field Pattern by Means of the Fourier Series

**Figure 9:**
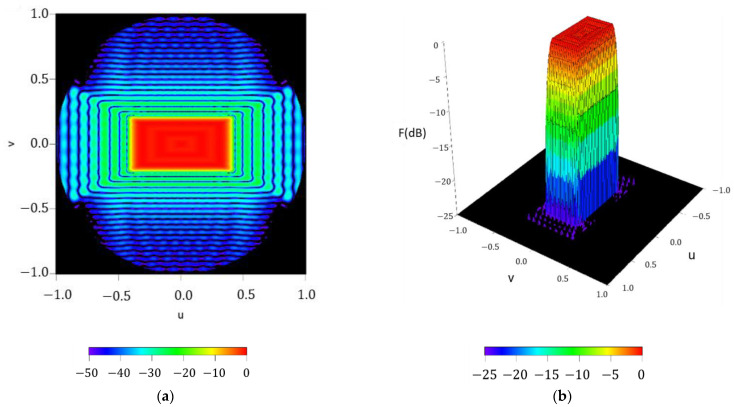
Reconstructed pattern of the required footprint for the case of a rectangular boundary of 20°×40° by means of the obtained Fourier coefficients in (2): (**a**) interpolated image with a threshold of −50 dB (**b**) surface plot with a threshold level of −25 dB.

As initial check for the performance of the methodology, the radiation pattern F(u,φ) reconstructed by means off the Fourier series with $N_{\max}^{F}=50$ is reported in Figure 9, as well as for the square contoured beam. Here, a pattern with a ripple level of ±0.53 dB and SLL =− 24.02 dB is obtained. Again, it is worth highlighting that also in this example the pattern nulls respect the rectangular shape imposed by the design, something which supports the precision of the method for reconstructing the footprint.

#### 3.2.2. Convergency Study of the Results. Performance of the Method

**Figure 10:**
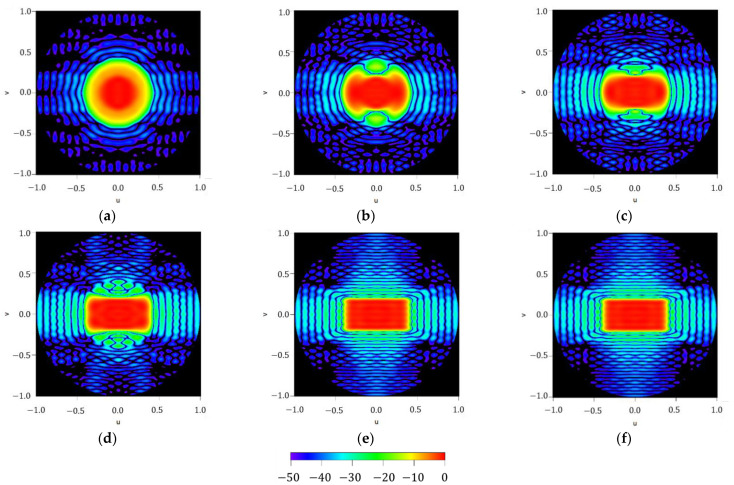
Figure series illustrating the iterative process devoted to generate a rectangular footprint by increasing the maximum order of the aperture distribution series $N_{\max}^{K}$ described in (7): (**a**) $N_{\max}^{K}=0,$ (**b**) $N_{\max}^{K}=2,$ (**c**) $N_{\max}^{K}=4,$ (**d**) $N_{\max}^{K}=8,$ (**e**) $N_{\max}^{K}=32,$ and (**f**) $N_{\max}^{K}=36$. All the interpolated plots described here have a threshold level set at −50 dB, for the normalized far-field power.

For illustrating the performance of the method in the case of a rectangular footprint of 20°×40°, different improvement steps obtained in the iterative process are shown in Figure 10. It is worth highlighting that in case of the rectangular boundary, the orders which represent a contribution different from zero are the ones proportional to two. It can be noted that, by adding more orders to the aperture distribution series, the technique improves the shape of the resulting pattern and, finally, it is capable of reproducing the required footprint (confirming, as well, the reasoning devised by means of the flowchart of Figure 1). Regarding the two final steps of the iterative process, SLLs of −22.65 dB and −22.79 dB are outlined, as well as the improvement in terms of ripple level falling from ±0.85 dB to ±0.83 dB.

#### 3.2.3. General Results of the Iterative Method

**Figure 11:**
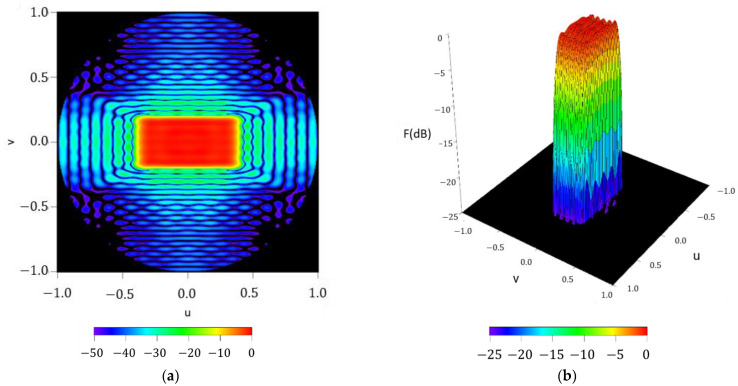
Reconstructed pattern for the case of a rectangular boundary of 20°×40° by means of a discretized array antenna: (**a**) interpolated image with a threshold level set at −50 dB (**b**) surface plot with a threshold level set at −25 dB.

In this section a description of the results of the iterative method is provided. In particular, the resulting pattern (reported in Figure 11) refers to a ripple level of ±0.83 dB, SLL=−22.79 dB, a dynamic range ratio of 289 and D=32.03 dBi. The resulting array antenna has a maximum radius of 12.5λ and a maximum order of 36 set for the aperture series. Additionally, in this case, as in Section 3.1.3, it is interesting to point out that the nulls presented in the antenna array pattern are arranged in a rectangular contour. Thus, these nulls are arranged following the same distribution of the nulls in the main beam region, as well as in the reconstructed pattern by means of the Fourier series (Figure 9), confirming the behaviour of the pattern reconstructed in Section 3.2.1. These results prove the precision of the present methodology regarding a target pattern reconstruction.

Regarding comparisons with planar arrays obtained by conventional techniques, in front of the 1044 elements obtained by the method, 1200 elements can be reported in case of imposition of a rectangular boundary which fits the problem, and 1976 would represent the number of elements to address in the case of a circular boundary. Therefore, decreases regarding the percentage of elements of −11.67% and −47.17% can be, respectively, highlighted.

Additionally, by including an element factor of a center-fed dipole with a length of 0.495 λ and placed at a distance of λ/4 above a ground plane (see Figure 2), a slightly better level of performance can be highlighted. More precisely, a directivity level of 32.13 dBi (+0.10 dBi), an SLL of −22.03 dB (−0.07 dB), and a ripple of ±0.70 dB (−0.01 dB) are reported.

**Figure 12:**
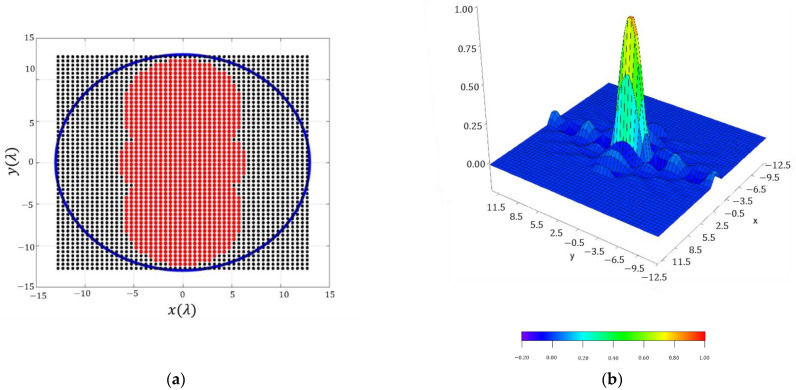
Discretized array antenna which generates the pattern of Figure 11: (**a**) antenna array configuration (the red dots represent the elements switched ON) within a rectangular lattice and for a maximum radius of 12.5 λ. (**b**) Normalized excitation currents of the array antenna.

In this framework, the layout of the antenna and the obtained shape of the distribution are reported in Figure 12, where its amplitude values are shown. In this case, the obtained distribution is pure-real, since a pure-real far field pattern was used as an input of the method.

If an improvement in terms of dynamic range ratio is performed, by selecting the aperture excitation currents with a level lower than 0.02, a dynamic range ratio of 49.95 is obtained. In this manner, the number of elements is decreased from 1044 to 916 and a directivity at broadside of 32.03 dBi is obtained.

### 3.3. Numerical Application: Footprint for Covering Europe

In this test case, the application of the methodology to a footprint of continental Europe is proposed. As is reflected in [19,20,21,26], these requirements were obtained based on a specification for a geostationary satellite (more concretely the EuTELSAT footprint) located at $46^{\circ }N,\;10^{\circ }\;E$. The requirements are based on SLL=−25 dB and a ripple level of ±0.5 dB. To this aim, a discretization in a rectangular lattice of half-wavelength in both dimensions was proposed in the same line of previous examples. Differences in comparison with previous examples can be highlighted due to the lack of quadrantal symmetry for this numerical case of application. As can be seen from the results included in Section 3.1 and Section 3.2, pure-real excitations are obtained since symmetric footprints were addressed. Otherwise, for the present case of reproducing a contour compatible with the footprint necessary for illuminating continental Europe, the results obtained are characterized by means of a general complex aperture distribution. In such a way, both amplitudes and phases in the elements’ currents can be expected.

#### 3.3.1. Reconstruction of the Far-Field Pattern by Means of the Fourier Series

**Figure 13:**
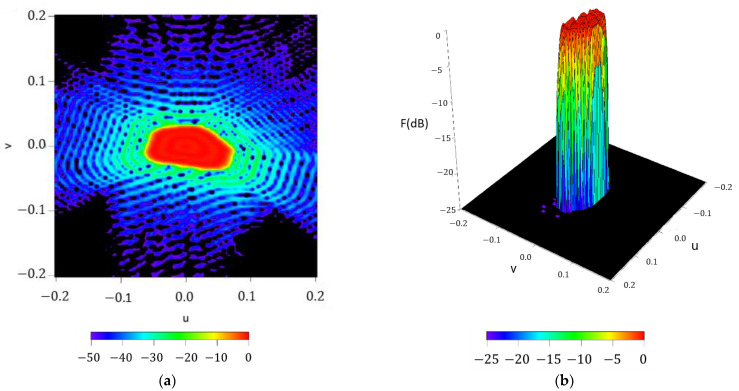
Reconstructed pattern of the required footprint for the case of the continental Europe footprint by means of the obtained Fourier coefficients in (2): (**a**) interpolated image with a threshold level set at −50 dB; (**b**) surface plot with a threshold level set at −25 dB.

As an initial check of the performance of the methodology, the radiation pattern F(u,φ), reconstructed by a Fourier series with $N_{\max}^{F}=50$, is reported in Figure 13, in the same manner as in previous sections. In this case, a pattern with a ripple level of ±0.525 dB and SLL = −24.54 dB is obtained. In order to appreciate the shape of the main beam, a zoomed image of the u and v axes is provided, analyzing a region between −0.2 and 0.2. It is worth highlighting that, in this framework, the Earth illumination for a geostationary satellite (such as the EuTELSAT) occurs in a cone defined by the angles ±8.3°. Thus, the zone of the pattern not shown here misses the Earth and is inconsequential.

#### 3.3.2. Convergency Study of the Results. Performance of the Method

**Figure 14:**
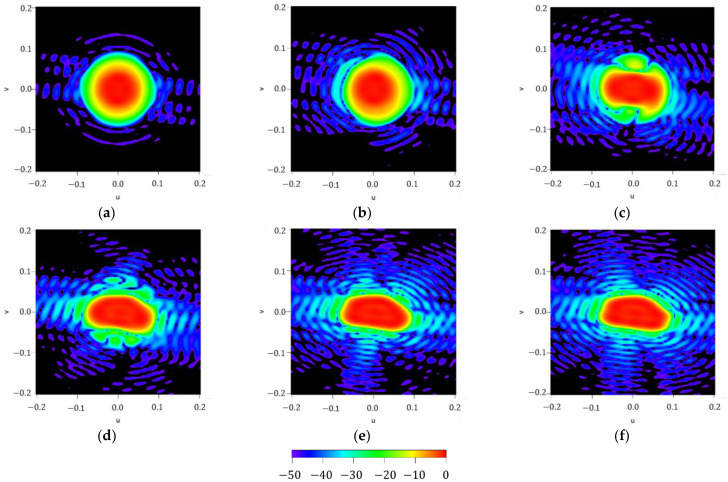
Figure series illustrating the iterative process devoted to generating a rectangular footprint by increasing the maximum order of the aperture distribution series described in (7): (**a**) $N_{\max}^{K}=0,$ (**b**) $N_{\max}^{K}=1,$ (**c**) $N_{\max}^{K}=2,$ (**d**) $N_{\max}^{K}=5,$ (**e**) $N_{\max}^{K}=10,$ and (**f**) $N_{\max}^{K}=28$. All the interpolated plots here described have a threshold level set at −50 dB for the normalized far-field power.

To also illustrate the performance of the method for the continental Europe footprint, different improvement steps obtained in the iterative process (described in Figure 1) are shown in Figure 14. It is worth highlighting that, in case of this irregular shape of the footprint, each one of the orders of the coefficients of aperture distributions presents a contribution different from zero. Then, the addition of more orders to this series improves the shape of the resulting pattern to finally reproduce the required footprint (as is explained in the flowchart reported in Figure 1). Regarding the two final examples of the iterative process, SLLs of −22.36 dB and −22.68 dB are reported, as well as the ripple level falling from ±0.75 dB to ±0.735 dB.

#### 3.3.3. General Results of the Iterative Method

**Figure 15:**
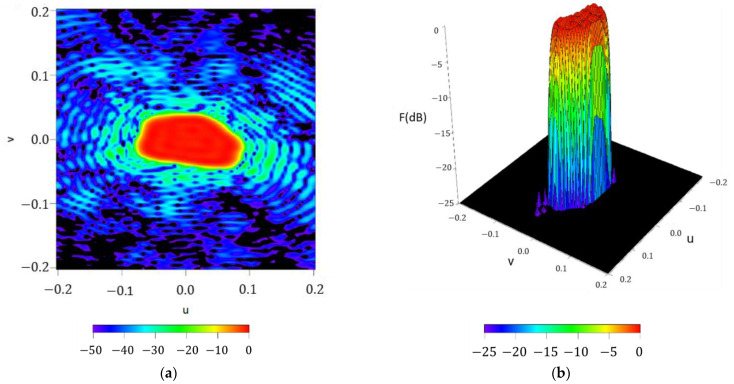
Reconstructed pattern for the case of the continental Europe footprint covered by the EuTELSAT (European Telecommunications Satellite Organization) satellite by means of a discretized array antenna: (**a**) interpolated image with a threshold level set at −50 dB;(**b**) surface plot with a threshold level set at −25 dB.

Thus, following the iterative method illustrated in the previous subsection, the resulting pattern (reported in Figure 15) refers to a ripple level of ±0.735 dB, SLL=−22.68 dB, a dynamic range ratio of 9978 and D=31.29 dBi. The resulting array antenna has a maximum radius of 82λ and it presents 44,452 array elements, and the aperture distribution series needs 28 orders for convergency $\left(N_{\max}^{K}=28\right).$ Additionally, in this case, as in Section 3.1.3, it is interesting to point out that the nulls presented in the antenna array pattern are arranged in a contour which fits the shape described by the imposition of the continental Europe zone of coverage. Thus, these nulls are arranged following the same distribution of the nulls in the main beam region as well as in the reconstructed pattern by means of the Fourier series (Figure 13), confirming the behavior of the pattern reconstructed in Section 3.2.1. These results prove the precision of the present methodology regarding a target pattern reconstruction, since it is composed by shrunk and stretched patterns for each angular cut. Additionally, if a rectangular array is analyzed, the number of elements would rise up to 57,528. Thus, in such a way, the number of elements is here reduced by −22.73%. By implementing a circular array which contains all the radii needed to reproduce the footprint, 82,452 elements are outlined. In this manner, the number of elements reported by the present method represents −46.09%.

#### 3.3.4. Subarraying: Performance of the Resulting Antenna

Then, in order to improve the results in terms of element number and dynamic range ratio, a strategy devoted to deal with subarray architectures is performed. To this aim, the subarray inclusion in the model was conducted through the expression of the array factor as in [30].

(10) $${AF}_{sub}(\theta ,\varphi )=f_{sub}\left(\theta ,\varphi \right)\cdot F(\theta ,\varphi ),$$

where the subarray factor is expressed as

(11) fsub(θ,φ)=sin(M2·ψx(θ,φ))·sin(N2·ψy(θ,φ))sin(12·ψx(θ,φ))·sin(12·ψy(θ,φ)),

where $\psi_{x}=2\pi d_{x}^{sub}\sin\theta \cos\varphi$ and $\psi_{y}=2\pi d_{y}^{sub}\sin\theta \sin\varphi .$ At the same time, $d_{x}^{sub}$ and $d_{y}^{sub}$ are the spacings (along the *x*-axis and the *y*-axis, respectively) between the internal elements of the subarray.

**Figure 16:**
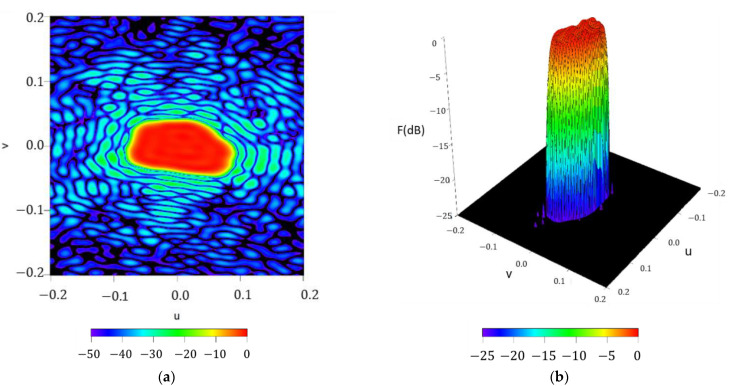
Reconstructed pattern of the required footprint for the case of illuminating continental Europe by means of the discretized array antenna in presence of 5 × 5 subarrays: (**a**) interpolated image with a threshold level set at −50 dB; (**b**) surface plot with a threshold level set at −25 dB. The pattern is zoomed in on the region defined by u=[−0.2,0.2] and v=[−0.2,0.2].

On this basis, different subarray architectures were addressed with arrangements of 2 × 2, 4 × 4 and 5 × 5 elements, respectively. In all of these cases the directivity level at broadside was kept at about 31.3 dBi (31.23 dBi, 31.17 dBi and 31.33 dBi, respectively), while the ripple level and SLL in the region of interest (illumination of the Earth) are in line with the values of the non-subarrayed case (−21.05 dB, −21.29 dB and −20.95 dB). On the other hand, an impact in terms of undesired lobes (i.e., grating lobes promoted by the use of subarrays) can be highlighted, where for the case of 2 × 2 subarrays, SLL_out_ of −19.39 dB is used, while the reported values for the 4 × 4 and 5 × 5 examples are −15.57 dB and −13.54 dB, respectively. These undesired lobes are provoked by the arrangement of the different subarrays present on the antenna. Since this spacing between these subarray structures is more than half of the wavelength, it causes the appearance of undesired high lobes in the zone of side lobes because of the periodicity of the above-mentioned structures. Thus, the resulting pattern of the example with subarrays of 5 × 5 elements is reported in Figure 16. Here, the performance of the pattern is reported in the region of interest regarding geostationary applications.

**Figure 17:**
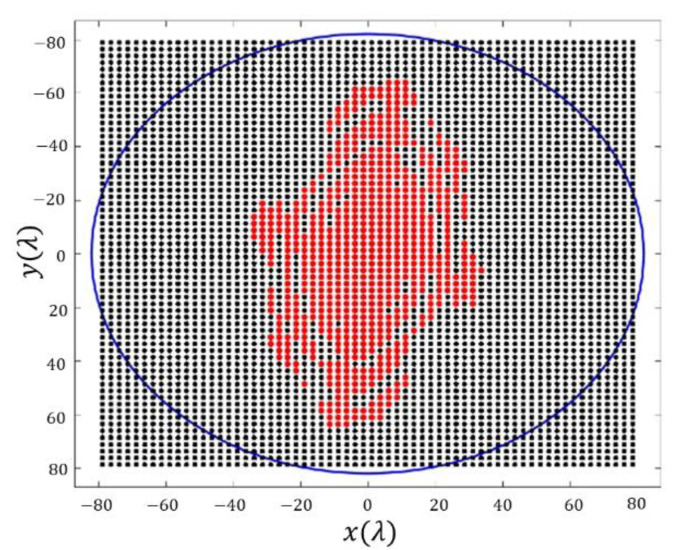
Layout of the antenna array with an inclusion of 5 × 5-element subarrays and by deleting the low-excited elements ($|I{|}_{max}/{\left|I\right|}_{min}$ = 46.81). Final number of array elements: 695.

Additionally, the array layout after eliminating the low-excited elements and adjusting the dynamic range ratio to 46.81 is shown in Figure 17. In this manner, a planar array of 695 subarrays is obtained and it reconstructs an acceptable footprint for illuminating continental Europe, since a directivity value of 31.33 dBi is obtained at broadside. In the other two cases (2 × 2 and 4 × 4), the obtained level of directivity at broadside was 31.23 dBi and 31.17 dBi, respectively. In these cases, a number of subarrays of 4365 and 1080 were achieved by means of setting dynamic range ratios of no more than 50.

In order to observe the impact of a real element within the array framework, by means of the inclusion of the element factor for the scenario sketched in Figure 2, values of directivity of 31.32 dBi (+0.09 dBi), 31.29 dBi (+0.12 dBi) and 31.42 dBi (+0.09 dBi) are reported in the cases of subarrays 2 × 2, 4 × 4, and 5 × 5, respectively. In this same way, SLL values of −21.14 dB (−0.09 dB), −21.34 dB (−0.05 dB), and −21.05 dB (−0.10 dB) and ripple levels of ±0.83 dB (−0.01 dB), ±0.84 dB (−0.02 dB), and ±0.93 dB (−0.01 dB) are obtained. It is worth mentioning that the biggest impact on the resulting radiation pattern provoked by the introduction of the real dipoles over a ground plane is on its grating lobes. In such a way, values of SLL_out_ −22.05 dB (−2.66 dB), −16.87 dB (−1.30 dB), and −14.26 dB (−0.72 dB) are seen. Therefore, it can be confirmed that the introduction of the element factor alleviates the collateral effects of the presence of the grating lobe produced by the subarray strategy.

**Figure 18:**
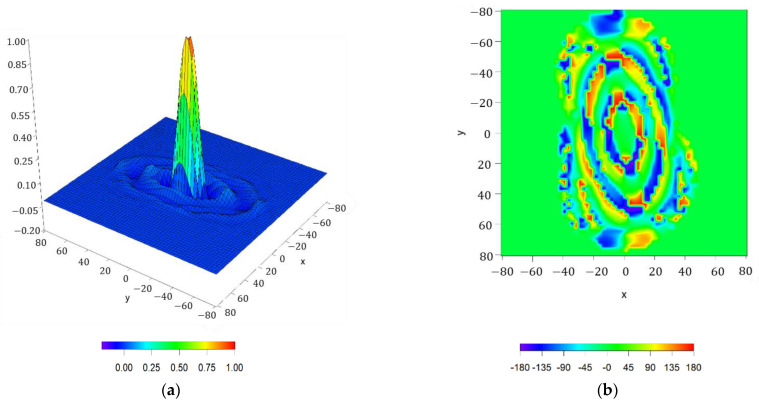
Discretized array antenna which generates the pattern of Figure 16: (**a**) normalized current distribution of the array antenna in the presence of subarrays of 5 × 5 elements. (**b**) Phases (°) of the antenna excitation currents.

In order to show the performance of the improved array antenna, the current distribution for this case is shown in Figure 18. Here, it can be noted how the aperture distribution, and therefore the resulting discrete currents, are complex (as it was introduced at the initial part of the present subsection). The motivation of this performance is based on the asymmetry of the case under test.

The use of subarrays, although it represents an alternative to keep the directivity at an acceptable level, has to be implemented with certain attention due to the appearance of grating lobes (the larger the spacing among subarrays is, the greater number of grating lobes appear in the radiation pattern). However, in the particular case of analysis of a geostationary satellite, moderately high energy losses appear at angular levels without real impact (more precisely, it happens at angular levels of more than $\pm 8.3^{\circ }$, i.e., angles of incidence falling out of the Earth). Thus, although these arrangements of elements offer the already-mentioned advantages, it is necessary to consider that they have an impact on the radiation efficiency of the array.

## 4. Discussion

In the present paper, an iterative methodology for reproducing an arbitrary footprint by means of a planar array of radiating elements was devised. A few iterations were necessary in order to permit the convergence of the present method. In this framework, subarray arrangements and improvements in terms of dynamic range ratio by eliminating low excited elements were addressed. It is worth highlighting that, in the present work, rectangular grid arrays were proposed, based on their practical application from a feeding point of view. Therefore, as is well-known, limitations on the methodology can be outlined due to this discretization strategy in a rectangular lattice. Furthermore, by highlighting the necessity of preventing the presence of a distribution with a huge variability, it is worth mentioning that, for reproducing a footprint without suffering these types of problems, not more than two ripple cycles in the pattern could be established as a good compromise. It is also well known that a distribution which produces such kind of patterns presents this variability. Thus, based on the results depicted here, the present methodology overcomes techniques of the state-of-the-art for reproducing arbitrary footprints presented in the previous literature, since it refines the method envisaged in [2] for developed efficient planar arrays in terms of number of elements. This improvement is based on the lack of assumption regarding symmetry on the antenna array pattern and/or simplicity of the continuous aperture distribution (intended as a Fourier of the entire three-dimensional target pattern).

More particularly, for the cases of study present in this paper, it is worth highlighting the necessities of the method to deal with footprint patterns with shapes that present an extreme difference between perpendicular axes. More precisely, the example of the rectangular boundary represents a more challenging scenario for the procedure than the square contour, because the method (for instance, the iterative steps illustrated in Figure 10) has to first mitigate the zones of the initial circular footprint (order zero approximation) in order to fit the required shape of the rectangle. In this manner, a greater number of iterative steps (i.e., Fourier coefficient of higher order) is required in comparison with cases as the footprint of the square contoured beam.

Regarding computational costs of the methodology, the most expensive test case aimed at being involved within the procedure is represented by the footprint of continental Europe, as an application of the EuTELSAT geostationary satellite. In this particular example, the time costs of producing the different coefficients of the series and discretizing the planar array for a maximum order of $N_{\max}^{K}=50$ is about 8 min and 35 s. Once the table of coefficients (both $F_{n}$ and $K_{n}$) has been determined and the model is calibrated, a maximum time of computation (i.e., assuming $N_{\max}^{F}=N_{\max}^{K}=50$) less than 51 s for each iteration can be reported. In such a way, we are introducing a technique which is computer-efficient, inexpensive, and rapidly convergent. The simulations described in this work were developed by implementing the method described in the materials and method section in a MATLAB code and run in an Intel Corei7 machine (CPU model 4510U) at 2.60 GHz with 6 GB of RAM memory. As a future trend, analogous studies to the research reported in [14] can be proposed by introducing the present methodology within a phase-only synthesis. Since the technique envisaged in [14] would be integrated by an optimization with restrictions, its analysis falls out of the scope of the present paper.

## Data Availability

Data is contained within the article.
